# A Case Study on the Persistence of Swidden Agriculture in the Context of Post-2015 Anti-Haze Regulation in West-Kalimantan

**DOI:** 10.1007/s10745-018-9969-y

**Published:** 2018-02-27

**Authors:** Paul Hasan Thung

**Affiliations:** Department of Anthropology, Brunel University, Uxbridge, Middlesex UB8 3PH UK

**Keywords:** Swidden agriculture, West Kalimantan, Environmental Policy, Southeast Asian Haze crisis, Political Ecology

## Abstract

This case study analyses a ban on the use of fire in a district of West-Kalimantan in response to the 2015 Southeast Asian Haze crisis. Based on stakeholder interviews and participant observation, I address the dilemmas encountered at the district and village level as a result of transnational environmental politics. A stark example of a wider tendency for policies to restrict swidden agriculture, the case study provides insight into the persistence of swidden*.* Contradictions between different stakeholders’ experiences and understandings of local human ecology and haze politics ultimately rendered the ban ineffective. Future efforts at regulating fire in smallholder agriculture would therefore benefit from a clearer understanding of the relationships between fire, subsistence, and haze.

## Introduction

Overviews of where and how swidden agriculture^1^ is practiced currently report a rapid decrease in the extent of swidden in Southeast Asia (Padoch *et al.* 2007; Schmidt-Vogt *et al*. 2009; Van Vliet *et al*. 2012; Li *et al*. 2014). This trend is explained as a consequence of a combination of demographic, economic, social, political, and biophysical drivers (Van Vliet *et al*. 2012: 422; cf. Cramb *et al*. 2009; Fox *et al.* 2009). Public policies, which often reflect governments’ intentions to reduce and eliminate the practice of shifting cultivation (Fox 2000: 4), are identified as highly significant in pushing swidden farmers towards different livelihoods or from the land, either directly through eradication efforts and bans or indirectly through limitations on the recognition of ownership in shifting cultivation and by pushing for agricultural intensification and commodification (Dressler *et al*. 2017: 14 of 20; cf. Cramb *et al.* 2009: 328; Fox *et al.* 2009: 319; Ellen 2012; Van Vliet *et al.* 2012: 422). This is cause for concern not only because the well-being of the swidden farmers is often negatively affected but also because the land uses that typically replace shifting cultivation perform relatively poorly in terms biodiversity, carbon sequestration, and other ecosystem services (Fox 2000; Bruun *et al*. 2009; Rerkasem *et al*. 2009; Padoch and Pinedo-Vasquez 2010; Labrière *et al*. 2015; Dressler *et al*. 2017).

With an abundance of factors driving this demise, the opposite question arises of why swidden agriculture persists in large areas of the tropics. Research points at its adaptability, cultural importance, importance for food security, as well as continued environmental, economic, and technological constraints to intensification (Cramb *et al.* 2009; Ellen 2012; Van Vliet *et al.* 2013). However, there have been few explanations of the tension between the persistence of swidden agriculture and attempts to outlaw it. Literature on the *demise* of shifting cultivation identifies bans as a cause of swidden decline but leaves unanswered the question of how, in areas where a ban is formally present, swidden often persists. Conversely, most studies on the *persistence* of shifting cultivation are in situations where it is not illegal but strongly discouraged (e.g., Laney and Turner 2015).

Here I directly address the tension between swidden and policy through a study of the persistence of swidden agriculture in Kapuas Hulu, West Kalimantan, in the weeks directly following a decree that rendered it illegal. The decree, issued by the district police on 13 July 2016, was an effort to prevent a repetition of 2015 when severe air pollution from fires in Indonesia damaged health and economies in multiple Southeast Asian countries (Balch 2015; Tacconi 2016). It stipulated sentences of three to ten years in jail plus a fine of IDR 15bn (over 1.1 million USD) to all who set fire to forests, farmlands, and gardens in the district of Kapuas Hulu, West Kalimantan (MB 01 VII 2016). At first glance, this regulation made it impossible to continue practicing swidden agriculture. The case study explains how and why it failed to do so.

Pivotal to understanding the persistence of swidden agriculture is the realisation that “[t]he discrepancy between rule and reality is one of the most striking characteristics of shifting cultivation policies” (Van der Ploeg and Persoon 2017: 72). There is great variation in the extent to which policies are supported and implemented on the local level, so that an examination of their impact on swidden cultivators needs “to look beyond the mere letter of forest [and other] policies pertaining to shifting cultivation […]” (Van Der Ploeg and Persoon 2017: 72). To achieve such vision, I focus on the ‘politics of swidden,’ a term borrowed from Pham *et al*. (forthcoming), which I use here to indicate the complex of policy “interpretation, accommodation, negotiation and resistance” (Cramb *et al*. 2009: 328).

Research accordingly focused on how different stakeholders on the district and village level interpreted and positioned themselves in relation to the ban. Research activities consisted of semi-structured stakeholder interviews (12), informal interviews with villagers (15), and participant observation (14 days). Six stakeholder groups were identified: the district government, district police, indigenous rights NGOs, environmental NGOs, the village government, and villagers without a government position. Participant observation took place in a village located on a minor river branch in the headwaters of the Kapuas river, home to a dominantly Catholic community of 150 registered households, practically all of whom practice swidden cultivation, and most of whom identify ethnically as *Dayak Tamambaloh.* Data from household surveys and focus group discussions conducted in the same study village a few months earlier by the Centre for International Forestry Research (CIFOR) was useful for verifying information on livelihoods and land use change.

The results reveal that the ban should be understood as an outcome of the dilemmas that transnational environmental politics impose upon local actors. Formal support for the ban was determined not so much by how stakeholders interpret the local human ecology, but primarily by their interpretation of what was politically required. The 2015 Southeast Asian Haze crisis created an atmosphere of political urgency. Stakeholders who held a position within Indonesia’s political hierarchy felt that their support for the ban was necessary to show their political superiors their commitment to preventing another disaster. Nevertheless, they were aware of strong arguments against the desirability and feasibility of the ban on the local level. A shared understanding of the importance of swidden agriculture for subsistence and the limitations of state power in the illegible landscape led actors to act in mutually accommodating ways. Building on these shared understandings, swidden farmers found a “way out” through negotiation and non-confrontational forms of disobedience – resolving, at least temporarily, the tension between local human ecology and transnational political expediency.

## The Politics of Swidden

This analysis builds on theories of peasant politics as well as the results of previous research on the persistence of shifting cultivation in the context of strong anti-swidden policy in Madagascar and Vietnam. Christian Kull’s (2002) paper on fire use and regulation in Madagascar describes how peasants have for a century evaded state sanctions on burning land by ‘taking advantage’ of three factors. First, the natural characteristics of fire are such that its use for clearing land can be masked, because it “does not depend upon humans for ignition, it is self-propagating and can do its work in the absence of people, it is easily lit anonymously, it can accomplish multiple purposes simultaneously, and the link between cause and effect is rarely straightforward or predictable” (Kull 2002: 10). Second, the allocation of responsibility is further complicated by village solidarity vis-à-vis the state. Motivated by the perceived legitimacy of fire and a desire to avoid being governed by an outside authority, villagers upheld a moral code of not testifying against one another. Third, the peasants took advantage of the state’s limited reach, internal diversity, and moments of distraction.

The theme of the internal diversity of the state is explored from the perspective of the state by Pham *et al.* (forthcoming), who draw on a multi-level government survey to show that actors on different levels of Vietnamese government approach swidden differently. Whereas people within the national government blamed swidden for deforestation and initiated programmes to eradicate it, district governments tolerated swidden because the farmers were located in border areas and could be relied upon to maintain national security.

Additionally political scientist cum anthropologist James C. Scott uses several useful concepts. First, he proposes that a *subsistence ethic* explains the “indignation and rage” (Scott 1977: 3) that motivated the failed peasant rebellions in Southeast Asia in the 1930s. Because peasant life is so precarious, peasants prioritise acquiring the minimum resources required for survival and believe that everyone has a right to these resources (ibid., 55, 167). Scott argues that the commoditisation of agriculture was unacceptable to peasants because it eliminated institutions that increased subsistence security (ibid., 168–189). In parallel, I argue here that subsistence ethics undermine policies that restrict swidden.

Second, the theory of *state optics* (Scott 1998) asserts that state-led development projects structurally fail because the state acts on models of the world that do not express the complexities of locally developed social and ecological systems. The logic of state optics and illegibility helps explain the shortcomings of the ban, revealing the structural challenges to state rule posed by shifting cultivation, “an especially complex and hence quite illegible form of agriculture from the perspective of a sovereign state and its extension agents” (Scott 1998: 283; cf. Colfer *et al*. 2015: 63; Ellen 2012: 23–24; Padoch *et al*. 2007).

Third, the concept of *everyday resistance* (Scott 1985) highlights resistance outside collective, delineated events such as rebellions and revolutions. Everyday resistance is characterised by a ‘quiet evasion’ rather than ‘open defiance’, and is often ‘masked with symbolic conformity’ to avoid retaliation (ibid., 32–33). Seemingly inconspicuous acts such as “foot dragging, dissimulation, desertion, false compliance, […], sabotage, and so on” (ibid., xvi), achieve substantial gains without provoking a clash. Scott developed the theory to characterise the intra-village social tensions resulting from the green revolution in rural Malaysia. Swidden cultivators can resist criminalisation using similar tactics.

Admittedly, Scott’s theories are limited when it comes to answering broader questions of state power and small-scale farming. Unacknowledged in the analytics of state optics, there are contested processes of state formation, non-state actors, co-optation of local knowledge, and the effects of failed projects (Li 2005). A focus on everyday resistance risks accepting as ‘givens’ what are more accurately perceived as effects of power, such as farmer conflict-aversion, and may unduly celebrate the effectiveness of everyday resistance (Mitchell 1990; Gutmann 1993). Finally, it cannot be assumed that farmers are primarily driven by a moral valuation of subsistence and not rational self-interest (Popkin 1980). These are genuine shortcomings, but they do not preclude insightful application of Scott’s theories to understanding swidden persistence.

## Positions and Interpretations

The limited sample of interviewees, ranging from one (the police) to four per stakeholder group, contained pronounced differences between groups, although the following presentation admittedly underplays heterogeneity within groups. The division between formally opposed and supportive groups cuts across a complex pattern of interpretative differences and similarities (Table 1). I first clarify the different interpretations of what could be called the human ecology of the ban, that is to say, how different groups conceived of the relations between humans and the environment in which the ban interfered. Second, I describe related political considerations. And third, I argue that local interpretations of what ‘the state’ sees led some to support the ban, while a subsistence ethic and landscape illegibility impeded effective enforcement.

**Table 1:** Overview of positions and interpretations regarding the ban

	Formal stance	Immediate necessity of fire for subsistence	Swidden responsible for haze?	Legal status of burning swidden	Explanation of the ban
District government	Opposed	Yes	No	[not discussed]	Political
Police	Supportive	Yes	Yes	Illegal	Political
Indigenous rights NGO	Opposed	Yes	No	Legal	Political
Environmental NGO	Opposed	Yes	Yes	Illegal	Political
Village government	Supportive	Yes	No	Illegal	Political
Villagers	Opposed	Yes	No	Legal	Political

## Subsistence, Fire and Haze

No interviewee disputed that swidden agriculture was necessary for the subsistence of a large part of the population in Kapuas Hulu. Alternative sources of income were not yet viable. Rubber prices were low, local wage labour opportunities were lacking, especially now that logging activity had decreased because of restrictive policy and depletion of valuable trees, and development projects provided only occasional work. Labour migration was not uncommon, most significantly to oil palm plantations in other parts of Kalimantan or logging concessions in Malaysia.

Government support for alternative forms of agriculture was still insufficient. Some located the problem in the ‘mindset’ of swidden farmers, because *sawahs* (irrigated rice fields) require a more precise and attentive approach than swiddens. Villagers themselves thought the practice of swidden agriculture was hard work and said they preferred alternatives, but a transition would require financial and technical assistance.

Conceivably, land and other assets could be sold for temporary subsistence. But selling assets is not usually seen as sustainable development for villagers, so none of the NGO, government, or police staff members mentioned it as a solution. Individual villagers did sell land to others within the village and a government official sold mineral extraction rights on his private land to an outside company. But collectively they opposed selling lands and minerals to outsiders such as oil palm companies because of concerns about environmental sustainability and the equitable distribution of gains.

Stakeholders also agreed that fire was integral to swidden. In general, the established use of fire in swidden was as follows. After cutting the vegetation and letting it dry, fire cleared the branches and leaves, creating space for crops and killing weeds. Fire simultaneously increased fertility of the otherwise acid soils. Without burning, rice plants might grow, but would not produce (*tidak berisi)*.

In contrast to the broad agreement on the connection between fire and subsistence, stakeholders disagreed on who was accountable for the haze crisis. The police and the environmental NGOs deemed the use of fire, which naturally contributed to haze, a sufficient sign of culpability. Villagers, indigenous rights NGOs, and district government officials disputed the problem definition for being simplistic. They countered that fire in swidden agriculture should be distinguished from fire used to clear peatlands for oil palm. They asserted that the use of fire on swidden went back to the ‘time of their ancestors’ (*dari nenek moyang*) and had always been controlled well. These fires were short-lived, small, and well contained. They said that the size of swidden plots in the village was typically below 1 or 2 ha, that villagers used fire breaks and sprayed water where necessary to stop spreading. At worst, it damaged a few meters of forest or some rubber trees – and customary law held the wrongdoers accountable. Moreover, shifting cultivators would only burn on mineral soils, so that fires lasted only a few hours. They didn’t burn peat, because peat was unsuitable for rice cultivation. In contrast, oil palm plantations were blamed with using fire on peatland, which can burn for weeks and is hard to contain or put out. In support, a district government official on August 11th pointed out there was already some haze in Pontianak while the swidden communities in Kapuas Hulu had not even started burning. The police and the environmental NGO deplored what they saw as the inability of swidden communities to understand the impact of fire on other people.

## The Politics of the Ban

The interviewees’ explanations of the ban were largely congruent but had little to do with local human ecology. Rather, the ban was understood in relation to national, transnational, and global haze politics as an outcome of political pressure to prevent future disaster. This pressure was described in terms of relationships of accountability, running from the villagers to the police and village government, up the political system to the president and from there back down toward the people of Indonesia and outward to other Southeast Asian countries affected by the haze, and, on account of greenhouse gas emissions, to the rest of the world. On each of these levels, the relevant actors had to prove that they were preventing haze. Given these pressures, the Indonesian president was eager to take rigorous measures, as reflected in his alleged statement that there should be “zero smoke” this year. No sources were provided to support that the president said this. The presidential instructions of October 25th 2015 (INPRES 11-2015) do not contain such a statement, but does contain a general call to “give firm penalties to individuals or legal entities that are involved in burning forests and lands” (INPRES 11-2015: PERTAMA: paragraph 4), but it’s debatable whether this should include swidden farmers.

While stakeholders agreed that the ban emerged in response to extra-local political forces, they disagreed on whether it was a necessary or good response. The police framed the ban as a direct implementation of the presidential instructions. They were furthermore worried about the president’s threat to fire local military and police chiefs if they were unable to control fires in their provinces (Soeriaatmadja 2016). Villagers agreed that the police had to enforce the ban “whether they want to or not.” However, the district government, the environmental NGOs, and the indigenous NGOs remained critical of the ban.

A district government official said that “concerning this fire policy, it is the government that is wrong, not the people, not necessarily the DINAS (district government), but the president who ordered that there should be zero smoke.” District officials blamed the president for having set “unrealistic goals,” which transfer the problem to lower levels of government. The police, furthermore, were blamed for not consulting with the district government departments before issuing the decree. The district forestry service had already implemented the presidential instructions by urging farmers not to burn, but in the form of an “invitation” (*ajakan*) instead of a prohibition (*larangan*). The district government officials reasoned in accordance with the subsistence ethic that their inability to provide sufficient alternatives restricted their authority to dictate the actions of the villagers: “Maybe for us what they do is not good, but they need to survive. We cannot give long-term alternatives; we have to give an alternative first.” This insight partly reflected the fact that some government officials had family members who practiced shifting cultivation. One official recalled not being able to defend the ban to his family.

Likewise, an environmental NGO staff member found the ban not comprehensive enough. It did not reflect an understanding of why people used fire. He took the opportunity to remind the government of their expertise in environmental sustainability, urging the government to support his NGO’s projects promoting alternative livelihoods, which would pave the way to a legitimate and effective ban by next year.

Indigenous NGOs used legal arguments to question the ban. Paragraph 1 of Article 69 of Law 32 of 2009 about the Protection and Management of the Living Environment (UU 32-2009) prohibits the use of fire to clear land, but Paragraph 2 provides an exemption to the cultivation of ‘local[crop] varieties’ on areas smaller than 2 ha per household that are managed in accordance with local wisdom, which includes a fire break. The indigenous NGOs maintained that this exception should be upheld in the efforts to fight haze. NGO staff had implored police officers not to prohibit the people from using fire, because it would be illegal. They had also presented their argument to the provincial government and national government, and found that the government as well as the police were open to reasonable discussion. The police, however, countered their legal argument by pointing at a regulation issued in 2010 by the Ministry of the Living Environment (PerMen 10-2010), which stipulates in Paragraph 3 of Article 4 that the exemption from the prohibition on burning mentioned in UU 32–2009 does not hold under conditions of below-normal rainfall, long droughts, or dry climates. It is not obvious which argument is legally correct, also because ministry regulations (*PerMen*) have lower legal status than laws (*Undang-Undang*).

The village government staff, although they did not think the ban was legitimate or purposeful, decided their best option was to give at least nominal support. Partly, this was a matter of resignation regarding the distribution of political power. They felt that they were not in a position to change the policy, because “the village government is the lowest government,” as one official put it. As they saw it, policy came already formulated from “above” or “the centre” and the village government merely implemented. They were also not in a position to *resist* the policy, because “to fight the police and army, that hurts. We do not have the power *(kemampuan)* to challenge the government.” The story of the head of a nearby village who had been arrested by the police after he gave villagers written permission to burn strengthened this fatalism. Frustration about this lack of power is evident in an official’s remark: “Smart people in Jakarta have defined this policy, they have thought about it – just right. Us ignorant farmers, what do we know?”

For others supporting the ban was a strategy to secure the flow of resources and attract aid. The village receives an increasing amount of development aid from the higher levels of government as well as from national NGOs and international donors. The disbursement of funds and assistance is seen to be conditional on the good conduct and compliance of the villagers and their administration. The implementation of development projects is especially uncertain. People do not know if and when extension workers will arrive at the village again. In this context, the village government used support for the ban on fire to attract aid. In particular, the village government hopes this year to secure the construction of *sawah* fields, which the central government has promised to do for many years.

## State Optics, Subsistence Ethics and Landscape

The analysis so far validates Li’s argument that states do not formulate coherent polices in the centre and impose them upon the peripheries (Li 2005: 384–385). Even though it was presented as such by the police and experienced as such in the study village, the ban on fire was not in fact formulated in Jakarta. The presidential instructions did not explicitly target shifting cultivators, and the ban was contested by multiple actor groups who took different positions on what the president’s instructions meant and what actions were required. But while attention to processes of ‘positioning’ indeed “brings a more complex field of meaning and action into view” (ibid.), it nonetheless provides further insight to also recognise the logic of state optics, which here works *through* the interpretation of local actors.

For the district police, issuing and supporting a blanket ban on fire was a good way to ensure that what the state saw reflected well on themselves. They mentioned that the central state saw satellite images of smoke as well as the reports from lower levels of government, the police, and others. A ban was likely to reduce the number of hot spots detected by satellite because it legitimised fire-extinguishing activities and acted as a deterrent on the use of fire. Perhaps more importantly, fire-extinguishing activities, expeditions to villages to inform people about the ban, and surveillance patrols made for persuasive evidence of haze fighting activity.

For the village government, to formally oppose the ban, as some village leaders in other villages are reported to have done, would have been a way of drawing attention to the needs of the villagers. But they would have had to argue on the basis a distinction between types of fire that might be lost to a state that is worrying about a transnational haze disaster. Therefore, supporting the ban was an equally sensible strategy. Superiors might reward an apparently compliant part of the political hierarchy. At the same time as these actors nominally supported the ban, its enforcement was strongly limited by their awareness of its incompatibility with subsistence and by the complexities of the landscape.

The tension is apparent to begin with, in the efforts of the police to inform the villagers about the ban, a process called ‘socialisation’ (*sosialisasi).* Following socialisation, the police took pictures of villagers holding banners that denounced burning, thus documenting evidence of popular understanding and support. But during socialisation, villagers often asked them for a ‘solution’ (*solusi)*: how were the people to sustain themselves without the use of fire? The police officer admitted: “We too are confused how to answer them.” They reported the issue to the district government’s department of agriculture, but continued socialisation activities. So while they produced images of successful socialisation, at the same time they acknowledged that they could not dismiss nor satisfy the villagers’ demands for a solution.

The police were cautious of the “indignation and rage” (Scott 1977: 3) that a denial of the right to subsistence might cause. The police officer spoke of a “dilemma:” “we want to uphold the law, but we also do not want a conflict with the people.” He indicated that 17 people had been taken to the police office for interrogation, but they had to be “very, very careful” (*sangat hati sekali*) because of potential “psychological impact.” If the communities feel attacked, “all of them will come here [to the police station], out of solidarity.” Until that day (August 26th), no small farmers had been jailed or fined over burning land in Kapuas Hulu or, as far as he knew, anywhere else in Indonesia. Nor had any farmer communities come to the police office to complain about the arrest of one of their members.

The restrictions of the landscape further limited the potential for enforcement. To track the fires, the ‘fire alert village platoons’ (*platon desa siaga api*) relied upon the coordinates of hot-spots on satellite images, as provided by ‘the centre,’ Jakarta. To reach fires was a challenge. As the fires were often located far from the road, it meant hours of walking carrying a water pump. The police lacked precise maps and it was often not practical to walk in a straight line from the road, because of the structure of paths, vegetation, and geological formations. Platoons would get lost. They sometimes decided not to go, when it was too far, taking into account that fires usually lasted only a short time. The challenge, curiously enough, was not to find the fires before they went out of control, but to reach them before they went out. On arrival, they needed a water source to connect to the pump. In the absence of water, they had to beat back the fire with sticks they found in the forest. This account is suggestive of strong limitations to the capacity of the police to control fire in remote areas, and supports the idea that their actions were mainly symbolic, or ‘ceremonial’ as an environmental NGO staff member phrased it. By being visible on the roads, coming to the fields and arresting perpetrators, they produced evidence of their commitment to fighting haze, although they were not willing or able to effectively stop swidden.

The village government also lacked authority and willingness to enforce the ban since there was no alternative way of providing food. They didn’t stop villagers from burning, as long as they did it far from the paved road. Much land away from the road was not officially registered as belonging to an individual household, so that it would be harder to pin the blame on someone. Additionally, the fire would be harder to spot and harder to reach, making it easier for the police and army to ignore. According to one village government official, burning close to the road would have forced the police to take action, as the police too must show that they are preventing haze. A burned patch next to the road would also have been hard to reconcile with the message of support for the policy that the village government was trying to convey. The village government accommodated the political pressure against fire with the local importance of fire. The policy could not be rejected nor could fire be avoided, so it was moved to where it was least obtrusive.

## “Ways out”

Before the first burn, villagers were uncertain how to respond to the ban. They felt trapped between the ban and the need to produce food, and talked about the need for “a way out.” Villagers didn’t feel like they were posing a threat to other people and felt no moral obligation to forego their practices. “[Only] if the government can feed us, can they prohibit us [from] burn[ing],” they would say in accordance with the subsistence ethic. And: “If there was *sawah*, we would not be doing this [shifting cultivation].” They did fear the threats of the police and army that there would be “no mercy,” but while preparing their plots they wondered: “If not burnt, then how?”

Ways out were a popular topic of debate. One extreme was the option of violent resistance, under the motto: “It is better to fight now than starve later.” The other extreme was to abandon swidden agriculture. On these lines, a village government official proposed to wait until the rice ran out, and then go to Putussibau, the regional capital, to complain – on the understanding that the physical presence of hungry villagers would powerfully demonstrate the hurtful effects of the ban. Neither extreme was popular. Rather, the “way out” villagers were looking for was a middle way, which both enabled swidden agriculture and prevented an open conflict. They were looking for tactics of non-confrontational disobedience.

The different tactics that were discussed in conversations with and among villagers exploited the limited capacity and willingness of the police and army to enforce the ban (Table 2). It would be hard for the police and army to track down and physically reach the fires in time to catch the perpetrators, the more so when fires were coordinated to occur on different days, small, quick, remote, and only accessible on foot. If, on untitled land, the fire had burnt out and there were no people present when the police and army arrived, it would be hard to find and punish the responsible household. It was furthermore anticipated that the police and army would be reluctant to enter village territory and catch the villagers because of the possibility of violent resistance: “The police do not dare to come here.” Stories of standoffs between other Dayak groups and the police and army strengthened this belief. According to one story, the Dayak Iban of a village down the road had burnt a plot right next to road, carrying BB guns with iron bullets, which they normally used for hunting. The police reportedly watched from a distance and did not dare to intervene. In another story, an older woman was caught burning her plot, but refused to come to the police station. In the uproar that followed, eventually people from two different villages came to support the woman and the police and/or army retreated. Villagers also reframed the prospect of jail as not undesirable, because it would mean free food. “If the government can afford to give me rice in jail, they are welcome to do so.” One man joked that he would bring his family and pets. Finally, the police, although forced to take action on haze, were reportedly not indifferent to arguments. Thus it was to some extent possible to negotiate.

**Table 2:** Possible responses to the ban as mentioned in discussions. The subsistence ethic moved villagers away from the left column, and tactics of non-confrontational obedience allowed villagers to stay away from the right column

Obedience	“Ways Out”	Conflict
Protest in district capital once food runs out.	Coordinate to prevent simultaneous fires	Fighting risks getting hurt Political confrontation risks losing political favour
	Choose remote locations	
	Choose untitled land	
	Use young and small fallows for quicker and smaller fires	
	Eat for free in jail (bring pets and family)	
	Burn after jail is full	
	Burn while the Iban fight the police	
	Rely on reputation of aggression to keep the police out	
	Negotiate with the police	

Although each of these strategies makes sense in its own way, very few of them were employed as such. Fields were burnt whenever the conditions were right, dependent on the previous clearing of vegetation, the weather conditions, and availability of labour, and simultaneous burning did occur. While the plots this year were reported to be smaller and more often on young fallows, this too was driven by practical rather than strategic considerations. Work on the plots had started late in the season, due to the timing of a ceremonial gathering. Smaller and younger fallows were preferred because they could be cleared more quickly. A diminished labour pool due to increased alternative activity in education, work migration, and local wage labour, as well as a diminished pool of eligible plots due to reduced willingness to farm far away from the location of permanent residence, provided additional practical reasons to choose small and young fallows. The reduced willingness to farm in remote locations, driven by the need to earn cash through additional wage labour as well as the need to live permanently in a location from where children could access education further meant that most of the chosen plots were not difficult to reach. On the other hand, it did appear that no fields were opened right next to the paved road. And there were reports of informal negotiations between villagers and police officers.

What is more, none of these strategies guaranteed a way out. Even when burning on a remote field the day after a good conversation with a police officer, it was conceivable that a group of zealous military men would come. In the face of uncertainty about how serious the threats of the police and military had to be taken, villagers proceeded with caution. They were not sure whether they should burn or not; they would decide once their fields were ready. A woman said: “I am still confused, but if one or two others burn [their plots], I will surely burn [my plot] as well.”

The discussions about different strategies did not, then, provide a fully worked out, agreed upon plan of action. Instead, each person adopted what might be characterized as a ‘proceed-and-see’ attitude, going through the preparatory phase of cutting the vegetation while interpreting the signs about if and how the ban was going to be enforced. Nevertheless, through such discussions, villagers encouraged each other to proceed. They were reassured that continuing shifting cultivation was the moral thing to do, that it was likely that the threat of punishment could not be substantiated, and that even if the police became serious about it, there were things they could do to minimize the risk. The decisive acts of everyday resistance were therefore (1) the discussions about a way out and (2) work on the still-legal phases of shifting cultivation, which prepared for (3) the tentative continuation of the use of fire.

## Conclusion

The central government did not as such plan to ban fire in shifting cultivation. It was an effect of the pressure on actors on the district level to prevent haze. The very agencies that implemented and supported the ban acknowledged that it could and should not be strictly enforced, but used it to show to their political superiors that they were preventing a crisis. This case highlights the potential risks of simplifying state vision and demonstrates a need for the central state to think and communicate about fire in swidden agriculture as a distinct category.

The effectiveness of the policy was undermined by a number of factors. While it is important to not overstate the importance of everyday resistance in general (Mitchell 1990; Gutmann 1993), the villagers resisted successfully, not in the form of outright defiance, but through a tacit, tentative continuation of their land use practices. Much like what Kull (2002) observed in Madagascar, they relied on a shared sense of morality and solidarity, natural characteristics of fire and landscape, and limitations to state capacity. Similar to the district-level tolerance of swidden described by Pham *et al*. (forthcoming), collaboration here extended beyond the communities that practice swidden agriculture to include district government and police officials. They collaborated to make the fires less visible to the central state and create an appearance of regulatory control. Whether actors agreed that swidden farmers had a right to subsistence, or merely feared an escalating conflict if farmer values were to be violated, the subsistence ethic was an important factor (*contra* Popkin 1980). Even the military and police did not attempt to enforce the ban at all costs, practicing tolerance where feasible and focusing efforts on larger fires nearer roads, which led a passer-by from a neighbouring village to remark that “there is only half a ban.”

On the basis of these interviews and observations, the ban on burning farmlands and forests in Kapuas Hulu appears ill-conceived and ineffective. As the fight against haze continues, the Indonesian administration’s struggle to implement and enforce regulation of peatland fire against entrenched economic interests will rightly take centre stage (Tacconi 2016; cf. Varkkey 2013). But the regulation of fire in smallholder agriculture remains equally tricky. In order to be legitimate and effective, such regulation should be backed by convincing data on the environmental impacts of smallholder fire and acknowledge the livelihood functions of local burning practices.

## Footnotes

1 ‘Swidden agriculture,’ used interchangeably with ‘shifting cultivation,’ is defined, following Mertz *et al*. (2009: 216) as “a land use system that employs a natural or improved fallow phase, which is longer than the cultivation phase of annual crops, sufficiently long to be dominated by woody vegetation, and cleared by means of fire.”
